# MR conditional prostate intervention systems and actuations review

**DOI:** 10.1177/09544119221136169

**Published:** 2022-12-01

**Authors:** Haipeng Liang, Zion Tsz Ho Tse

**Affiliations:** School of Engineering and Materials Science, Queen Mary University of London, London, UK

**Keywords:** MRI, pneumatic motor, MR-conditional, prostate intervention robot

## Abstract

Magnetic resonance imaging (MRI) has the ability to provide high-resolution images of soft tissues without the use of radiation. So much research has been focused on the development of actuators and robotic devices that can be used in the MRI environment so “real-time” images can be obtained during surgeries. With real-time guidance from MRI, robots can perform surgical procedures with high accuracy and through less invasive routes. This technique can also significantly reduce the operation time and simplify pre-surgical procedures. Therefore, research on robot-assisted MRI-guided prostate intervention has attracted a great deal of interest, and several successful clinical trials have been published in recent years, pointing to the great potential of this technology. However, the development of MRI-guided robots is still in the primary stage, and collaboration between researchers and commercial suppliers is still needed to improve such robot systems. This review presents an overview of MRI-guided prostate intervention devices and actuators. Additionally, the expected technical challenges and future advances in this field are discussed.

## Introduction

Prostate cancer is the second most common malignancy, after lung cancer, in males throughout the world, accounting for more than 240,000 new cases and approximately 34,000 deaths in 2020.^1^ The two most common procedures for screening men for prostate cancer are the prostate-specific antigen (PSA) blood test and the digital rectal exam (DRE). Transrectal ultrasound (TRUS) image guidance, where a needle is inserted into the prostate to obtain tissues for clinical analysis, is considered the “gold standard” for guiding prostate biopsies and interventions. The doctor physically inserts a TRUS probe into the rectum and pushes a biopsy needle through the rectum wall into the prostate gland under ultrasound guidance. A half-cylinder of tissue taken by the needle is pathologically tested to see if cancer is present. To improve the success rate of biopsy, several biopsy samples – typically six or more spatially distributed prostate core biopsies – are collected to provide representative samplings of the gland and identify the degree and extent of malignancy.^2^

The TRUS-guided biopsy has been widely used for its low cost, good real-time performance, and simplicity.^3^ However, the detection rate of TRUS-guided biopsy is only 20%–30%.^4,5^ Studies have shown that TRUS-guided prostate biopsy misses at least 20% of patients with cancer,^6,7^ meaning patients receive false-negative results.^8^ Therefore, the number of biopsy sessions has to be increased to improve the detection rate of prostate cancer, resulting in lots of repeat biopsy cases. Also, the risk of complications is increased. Despite developments in ultrasound imaging technologies, TRUS imaging is often unable to distinguish between healthy tissue and prostate abnormalities, so it is unable to identify or target lesions.^2^

Brachytherapy involves placing radioactive seeds into the prostate with high accuracy,^9^ which is normally performed using the transperineal approach, wherein the needle is inserted through the perineum, under the guidance of ultrasound. Both biopsy and brachytherapy procedures use two concentric shafts (an inner stylet and an outer hollow cannula) for obtaining tissues or implanting radioactive seeds into the prostate, so they can be carried out with the same device.^10^

Magnetic resonance imaging (MRI) is a widely used medical diagnostic tool.^11^ MRI-based medical devices have significant benefits over alternative imaging methods like CT and X-ray imaging. First, MRI is capable of producing high-quality images of soft tissues. Second, as three-dimensional imaging technology, MRI allows for the selection of any imaging plane in real time. Third, MRI does not expose patients or surgeons to ionizing radiation.^12^ So MRI can help clinicians navigate needles to interior organs and targeted lesions while taking biopsies or performing needle-based ablation techniques. For these reasons, MRI has become frequently employed in biopsies and seed brachytherapy,^13^ prostate interventions,^14^ and other interventional procedures.

The diameter of the bore of most major MRI scanners is around 60 cm. Due to the restricted space, operating in the bore is difficult for surgeons. As a result, the patient has to be transferred into the scanner for imaging and out of the scanner for needle insertion, which complicates the diagnosis and surgery process. Moreover, image registration is obtained based on 2D images, so the accuracy of a procedure relies on the surgeon’s experience.

In contrast to manual prostate intervention, the robot-assisted approach has several advantages. First, driven by precise components, the robot can reach the desired target and carry out the desired task more precisely. Additionally, a robot is capable of combining different procedures to reduce the operation time. Moreover, it is possible to monitor the manipulation of the robot in real time. With high-quality images of soft tissues, the spatial linkages between the lesion and surgical tools can be acquired, allowing robots to instruct surgeons to put surgical tools in targeted areas with great precision. For these reasons, the development of MR-guided robots has attracted much interest from researchers. Some such robots have already been adopted in clinical practice.

However, there are some challenges for robots working under MRI. First, because of the strong magnetic field generated by MRI, ferromagnetic materials in the MRI scanner can pose a risk. Besides, paramagnetic materials used in MRI scanners produce magnetic flux, which can distort the magnetic field of MRI, thereby lowering the image quality. For these reasons, medical devices that work under MRI must have non-magnetic material. As a result, traditional electric actuators based on electromagnetism cannot be used in MRI scanner rooms.^15^

Based on the response to the magnetic field created by MR scanners, two terms are introduced by the American Society for Testing and Materials (ASTM) in ASTM F2503-13: MR safe and MR conditional.^16^ MR safe devices pose no hazards under all MR environments, whereas MR conditional devices pose no known hazards within defined conditions.

Great effort has been put into the development of MR conditional robots. The UMCU robot developed by van den Bosch et al. in 2010^17^ was the first robot to insert a needle into a prostate gland under MRI. The robot has five degrees of freedom and is pneumatically and hydraulically driven, allowing the tapping part to be translated and rotated manually. With the guidance of a 1.5T MR bore, the robot successfully tapped a needle stepwise and planted several fiducial gold markers into the prostate.

Since then, several MR conditional and MR safe robot systems have been developed, showing the possibility of using robots under MRI.^10,18–20^ However, few of them reached clinical trial stages, and none of them received approval from the Food and Drug Administration (FDA) or other regulatory administrations.

In 2017, Johns Hopkins University reported a robot design with six degrees of freedom (DOF) motorized by six pneumatic motors.^21^ This robot successfully performed biopsies on five patients, and the design team is working toward the goal of receiving FDA approval. This work shows the great possibility for the adoption of MR robots in clinical practice.

For the intervention approach, the robot-assisted prostate intervention is classified into three different approaches: transrectal, transperineal, and transgluteal.

(1) Transrectal Approach:
  Several researchers reported MRI-guided systems based on the transrectal approach for prostate biopsy in Refs.^22–24^ Because no anesthesia is used during the procedure, the transrectal approach is advantageous for patients because it causes less pain compared with the transperineal approach.^25,26^ Furthermore, a longer distance to the prostate is required with the transperineal approach than with the transrectal approach, so needle deflection^27^ and small deviations during needle insertion may reduce the biopsy accuracy.^23^
(2) Transperineal Approach:
  In Refs.,^10,14,19,21,28^ MR conditional robot systems using the transperineal approach were presented. During brachytherapy, the perineum was employed for seed implantation. This is a preferable method for boosting the therapeutic ratio in brachytherapy.
(3) Transgluteal Approach:
  Transgluteal biopsies are performed in some robot studies^29,30^ because the transgluteal approach can reduce the risk of bladder, bowel, and iliac artery injury, and prevent intestinal germs from entering the prostate. However, local or general anesthetics are needed in this approach. Also, the long biopsy pathway magnifies any deformation of the needle, thus decreasing the biopsy accuracy.

Recently, many MR-guided systems have been developed using the transperineal approach because it carries a lower risk of sepsis and allows a greater proportion of peripheral zone to be accessed in comparison with the transrectal approach. Although the transrectal method is more commonly used by urologists, it has been considered to increase the risk of urinary tract infection and sepsis.^31^ In addition, compared with the transrectal approach, the transperineal approach detects a higher percentage of prostate cancer in the peripheral zone, where most prostate cancer is located.^19^ Furthermore, because of the increased risk of rectal injury and infection, the transrectal method cannot be utilized for therapy that requires many needle insertions.

As the number of MRI conditional robots with reportedly high accuracy has been increasing greatly in recent years, a large number of MR conditional actuators, including hydraulic, pneumatic, and piezoelectric motors, have also been created.

Piezoceramic actuators do not have magnetic components and are MR conditional, so they are often used in MR conditional robots. Piezoelectric motors have high power density because they directly transfer electric power into mechanical output. However, the electricity used to drive the motors can generate electromagnetic interferences (EMI), thus severely reducing the signal-to-noise ratio (SNR) and decreasing the quality of MR images.^32,33^ Therefore, radio frequency (RF) shielding is necessary, but it will complicate the system. Hydraulic actuators work in the same way as pneumatic actuators, but with an incompressible liquid instead of compressed air. As the liquid is incompressible, hydraulic actuators can provide a large power output and maintain a consistent dynamic output even with long fluid hoses. As a result, it is possible to keep an MR unsafe pump in the MRI control room and connect the motors to the pump through long hoses. Therefore, hydraulic motors could be another option for MR conditional robots. However, liquid leakage from hydraulic motors is a major concern because liquid leakage, no matter how little, is unacceptable in the clinical environment.^34,35^ Another concern is the inaccessibility of hydraulic pumps; unlike compressed air, the current utilization of hydraulic operations in hospitals is fairly limited, so clinical adoption would face an additional challenge.

The driving power of pneumatic actuators comes from compressed air, which has no effect on MR images. Components like driving parts, air hoses, and optical sensors can easily be made MR conditional, with no effect on MR images. Additionally, air leakage, unlike liquid leakage, poses no hazard to the clinical environment.^34^ Moreover, a compressed air supply is accessible in MRI scanner rooms, which can be used to drive pneumatic actuators. For these reasons, pneumatic actuators have become a more promising method for MR conditional robotics than piezoceramic and hydraulic actuators.

Optical sensors are sometimes used to supply precise position and speed control for pneumatic motors. These sensors can adjust air pressure and air pulses to regulate the rotation direction and speed. Additionally, optical sensors are commonly used for feedback to create a closed control loop,^36^ and specific proportional–integral–derivative (PID) and sliding mode control are needed for the position and speed control,^37,38^ all of these would complicate the system. Although stepper motors are controlled by air sequences, they can be operated step-by-step to achieve accurate position control without the use of any feedback components.

The first study of MR conditional actuators was published by Stoianovici et al. in 2007,^39^ and their actuator design has been used in many MR conditional robot designs.^21,40^ Since then, numerous other MR conditional pneumatic actuators have been presented.

Due to the advancement of 3D printing techniques, using a low-cost 3D print technique, MR conditional pneumatic actuators could now be fabricated easily. With the help of 3D printing or laser cutting techniques, specific design specifications can be adjusted according to the researchers’ requirement. Besides, by utilizing various types of reducers, the dynamic outputs of 3D-printed pneumatic motors can be customized to achieve different speeds and torques suitable for specific applications. Moreover, the use of 3D printing and laser cutting techniques makes it quicker and easier for researchers to fabricate prototypes to meet specific design requirements.

The operating principles, structural designs, and experimental performance of robot and actuator prototypes for MRI-guided prostate intervention reported mainly over the past decade are presented in this review. The current challenges in the development of MR conditional robots for prostate intervention and pneumatic actuators are presented as well.

## MRI-guided robots for prostate intervention

According to ASTM F2503, robots used in the MRI environment are classified as MR safe or MR conditional. The ASTM F2503 classification of some of the listed robots was not given. The use of nonmagnetic metal and piezoelectric motors can generate electricity and heat in the MR environment, so robots including these materials would most likely be classified as MR conditional. Although both have the possibility to be used under MRI, devices that meet the MR safe standard require less testing and can be used in a wider range of applications.

This review focuses on studies from the past decade. Table 1 lists some of the prostate intervention systems with the corresponding publication year, DOF, actuation method, accuracy, MR compatibility, and other features. Currently, considering the compact size, high positioning accuracy, and good dynamic performance, a variety of piezoelectric motors have been developed for commercial use. At the same time, many drivers are available for piezoelectric motors. As a result, a majority of robots for MR applications are powered by piezoelectric actuators. In this review, the first two robot systems from Johns Hopkins University using pneumatic motors are classified as MR safe. For the others, because of the use of piezoelectric motors or ultrasonic motors, the SNR reduction is significantly higher than the first two.

**Table 1.** List of MR conditional prostate intervention systems presented in the literature.

Reference	Year	Group	Degree ofFreedom(DOF)	Actuator	Accuracy	Signal-to-noiseratio (SNR)reduction	Feature
Fischeret al.^14^	2008	Johns Hopkins University	3	Pneumatic actuator	0.94 mm	5%	-Transperineal -Evaluated in phantom studies
Stoianoviciet al.^21^	2017	Johns Hopkins University	6	Pneumatic actuator	2.55 mm	MR safeby FDA	-Transperineal -Parallel-link mechanism -FDA approved -The biopsy time was recorded, indicating the system reduced biopsy time compared to manual approach
Kriegeret al.^18^	2013	Sentinelle Medical	3	Piezoelectric motor	3.7 mm	40%–60%	-Transrectal -Tested on phantom -Works with a passive 6-DOF arm
Su et al.^10^	2015	Philips Research North America	6	Piezoelectric actuator	0.87 mm	15%	-Transperineal -Customized driver and controller used -Phantom-based evaluation
Moreiraet al.^19^	2016	University of Twente	9	Piezoelectric motorsand a pneumatic actuator	1.84 mm	27%	-Transperineal -Parallel mechanism -Customized path planning algorithm -Each needle insertion took around 25 minutes
Moreiraet al.^41^	2021	Brigham and Women’s Hospital	4	Ultrasonic motors and Piezoelectric motors	2.4 mm	77%–85%	-Transperineal -Insertion trajectory analyzed -Needle insertion performed in vivo

Technically, two DOFs are required to place the needle guide in horizontal and vertical directions, and an extra DOF is applied to allow the needle to be inserted into the prostate with different trajectories for biopsy and brachytherapy. As a result, the robots proposed by Fischer et al.^14^ and Krieger et al.^18^ have three DOFs. However, with more DOFs, the robot can move and rotate along with the patients during surgery, reducing the influence of patient movement. Furthermore, the extra DOFs provide radiologists with more options for needle insertion trajectories. The robot developed by Moreira et al.^19^ has the most DOFs, allowing for great surgical flexibility.

### Transperineal prostate needle placement robot

**Figure 1.**
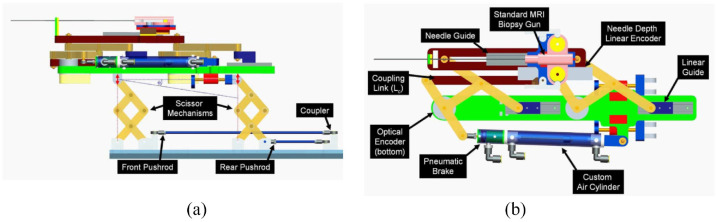
The mechanism of the robot^14^: (a) vertical view and (b) horizontal view.

A pneumatic transperineal prostate needle placement robot was created by Fischer et al. in 2008.^14^ The manipulator has a vertical and a horizontal motion. For the motion in the vertical plane, two scissor lift mechanisms are used in parallel to achieve 2-DOF motion, including vertical travel of 100 mm and elevation angle of 15°, shown in Figure 1(a). A second planar bar mechanism using two straight line motion devices called Scott–Russell mechanisms is applied to control the motion in the horizontal plane, shown in Figure 1(b). Pneumatic actuators are chosen as the driving parts due to their MR compatibility. A pneumatic brake is fitted for the purpose of keeping the position of the needle stable. Besides, the piezoelectric valves can be put into the MR scanner room, decreasing the lengths of tubes that connect the valves and motors. As a result, the response rate is improved. Optical sensors are used for positioning, considering their MR compatibility. A z-shape passive tracking fiducial^42^ is applied to build the transformation between the coordinate system of the robot and that of the MR images. Once the transformation is obtained, the position of the end-effector is determined by calculating the robot’s forward kinematics and encoder positions. The position of the end-effector in the image coordinate is then determined.

For the sake of MR compatibility, the controller placed inside the MRI scanner room is covered with an EMI shielded enclosure. All the valves and sensors are placed no further than 5 m away from the robot. The operation workflow, including registration, planning, targeting, monitoring, and verification, is shown in a customized graphical user interface (GUI).

An MRI test under 3T MRI showed that the average SNR loss was less than 5%, and the robot’s positioning accuracy was measured to be 0.94 mm. In the phantom study, the whole system workflow was assessed, successfully targeting five out of five 10 mm targets.

### APT MRI III system

**Figure 2.**
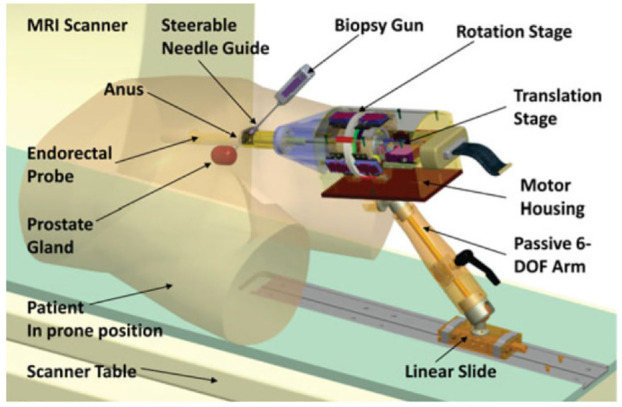
Computer aided design (CAD) drawing of the actuated robot for prostate intervention.^18^

**Figure 3.**
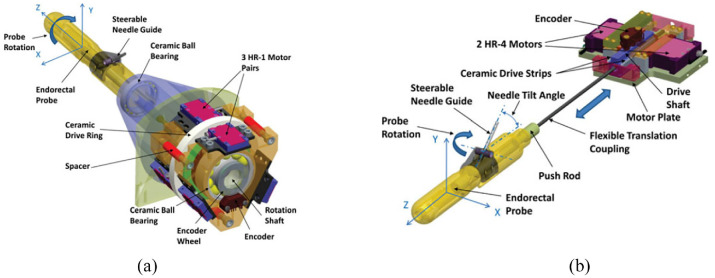
(a) CAD drawing of the rotation stage and (b) CAD drawing of the translation stage.^18^

Krieger et al. created an MR conditional robot for transrectal prostate intervention in 2013,^18^ shown in Figure 2. This robot, the APT MRI III system, has a similar mechanism design with the APT MRI system^43,44^ and APT MRI II system.^22,24^ Two actuated stages, powered by piezoceramic motors, are used to translate and rotate the needle guide. As shown in Figure 3(a), the axial rotation of the complete transrectal probe assembly is controlled by the rotation stage. As the MRI scanner bore has more axial space than radial space, the motors are mounted axially. Three pairs of HR-1 motors are equally spaced around the rotating shaft, generating a torque of 1.08 N·m to rotate the drive ring. The translation stage (Figure 3(b)) is actuated by two opposing HR-4 motors with an affordable linear implementation. The travel length is set to 28.7 mm to obtain a needle tilt angle range of 17.5°–40°. In addition, a 6-DOF passive arm with a sliding mechanism is created to hold the manipulator. Once the needle is positioned appropriately, the arm can be locked to resist a 30 N force applied at a distance of 200 mm. Then manual needle insertion is carried out.

Electro-optical encoders are applied to the robot for position tracking because of their high resolution and repeatability, and simple integration with a controller. One controller box consisting of one motion controller, two motor amplifiers, and an optical converter is placed inside the MRI scanner room.

An experiment showed that the SNR degradation was significantly reduced when RF shielding was used. Another experiment demonstrated that there was no SNR reduction when the motors were off, but an SNR reduction of 40%–60% was recorded when the motors were on. The maximum error of the robot was 3.7 mm in a study of seven MRI-guided biopsy needle insertions in a prostate phantom.

### A decoupling actuated interventional system

Su et al. developed a 6-DOF robot for transperineal prostate interventions in 2015.^10^ This design was improved in terms of structural stiffness, mechanical reliability, and simplicity of assembly over their previous work.^45^

**Figure 4.**
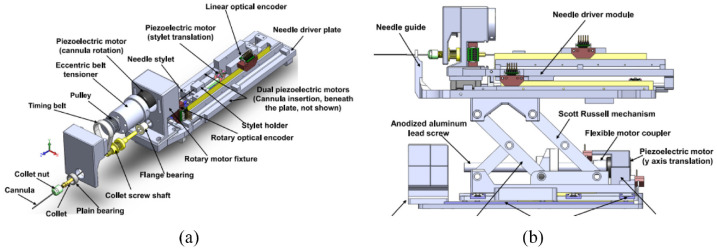
The two main modules of the robot^10^: (a) needle driver module and (b) Cartesian motion module.

This robot consists of two main structures: the needle driver module (Figure 4(a)) and the Cartesian motion module (Figure 4(b)). The Cartesian motion module provides three decoupled DOFs. A one-and-half Scott–Russell scissor mechanism driven by a lead screw is applied for the vertical motion. Two linear motors are used for the horizontal motion. The needle driver module is located on the Cartesian motion module and has three DOFs, including the translation and rotation of the cannula, and the translation of the stylet. All these motions are driven using piezoelectric motors.

A customized controller that integrates a microcontroller and a field-programmable gate array is developed to control the motion of the motors (both harmonic and nonharmonic motors) and manage the communication with the PC. Closed-loop control is provided by a combination of PI controller and commercial encoders. All the controllers and drivers are shielded in an aluminum case for the purpose of reducing image degradation.

In the registration, the robot coordinate relating to the patient coordinate is obtained by imaging the fiducial frame. Because the position of the needle tip can be calculated by the forward kinematic and optical encoder, the spatial links between the robot tip and the patient can be obtained. The robot kinematics are analyzed, revealing the position of the robot in relation to the fiducial frame.

The graphical user interface (GUI) of the robot includes the registration and calibration module, kinematics calculation, software control, and joint information display. The robot is registered with the patient coordinate system through Multislicebased fiducial registration.^46^ As a result, the needle tip position in the patient coordinate system can be obtained with an accuracy of 0.27 mm in translation and 0.16° in orientation.

An MRI test was performed with a Philips Achieva 3-T system, showing SNR loss was limited to 15%, and there was no obvious image interference. The root mean square error was 0.87 mm.

### MIRIAM robot

**Figure 5.**
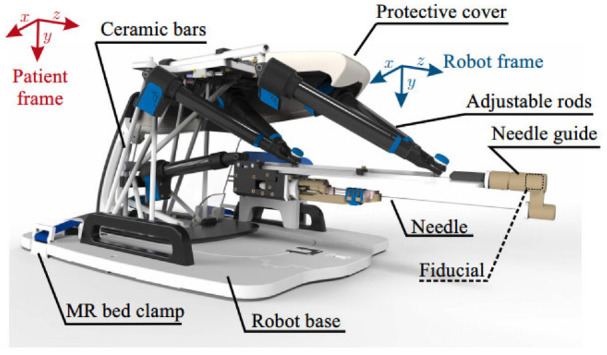
CAD drawing of the MIRIAM robot.^19^

The minimally invasive robotics in a magnetic resonance imaging environment (MIRIAM) robot with nine DOF, shown in Figure 5, was created by Moreira et al. in 2016.^19^ The robot consists of two main parts: a 5-DOF parallel robot and a 4-DOF needle driver. The parallel robot consists of five linear rods, allowing the robot to translate into three Cartesian axes and rotate around two axes to position the needle against the perineum. All linear motions are achieved with a combination of commercial optical encoders and piezoelectric motors, which are nonmagnetic. The needle driver is employed to insert, rotate, and fire the needle while in the procedure. The needle insertion and rotation are driven by piezoelectric motors, and the stylet is fired using pneumatic actuators.

In preoperative planning, the coordination relationship between the robot and the patient can be obtained by detecting the fiducial at the end of the needle with the MR image. The control system is located in the MRI control room for the requirement of MR compatibility. Rotation minimization algorithm (RMA) and Random path generator algorithm (RPG) are used to explore an optimized path to reach the target.

In an MR compatibility test, the maximum 27% SNR reduction was recorded while the motors were running, and the robot caused no visible image degradation. An average targeting error of 1.84 mm was shown in the needle steering experiments and was less than the insertion requirement. Besides, each needle insertion took around 25 min, which was less than in the manual procedure.^47^

In summary, the 9-DOF design gives the robot more flexibility and makes it possible to avoid physical obstacles during procedures. Because of the use of piezoelectric motors, the proposed robot is classified as MR conditional according to standard F2503-05.

### MrBot

MrBot, created by Stoianovici et al. in 2017,^21^ was designed for transperineal prostate intervention and was based on another robot structure.^40^ It has six DOFs and is driven by six MR safe pneumatic step motors (PneuStep^39^). Additionally, it does not contain any metallic or conductive material. It is recognized as MR safe, and was the first robot authorized by the FDA for use in an MR environment.

**Figure 6.**
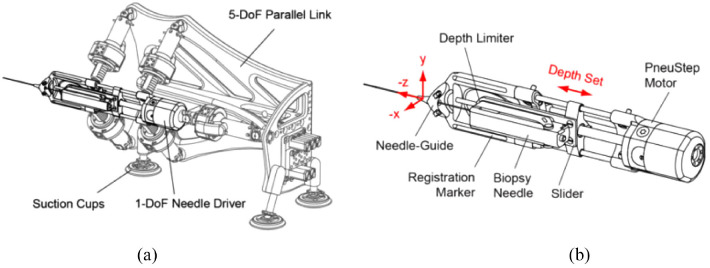
(a) Robot structure and (b) needle driver.^21^

This device has a 5-DOF structure (Figure 6(a)) and a 1-DOF needle driver (Figure 6(b)). The 5-DOF structure is driven by five linear actuators, allowing the needle to work in three axes of translation and two axes of rotation. The needle driver is placed on the top of the 5-DOF structure and is driven by a pneumatic motor with a screw lead. The entire actuated structure, excluding the needle guide, is covered with a sterile bag for sterilization requirements. To fulfill the requirement of the FDA, all materials in contact with humans are biocompatible, and commercial biopsy guns are applied.

The motion control card (MCC) is the core element in the controller. It receives feedback from each joint, including the encoder and limit switch signals, which are converted to electro signals. The targets can be registered in the robot coordinate system using the registration markers on the needle driver, with which the computer can obtain the target position of the needle guide. Then, the position of each motor can be calculated using inverse kinematics. With the target positions and orientations sent from the computer, the MCC sends the control signals to all the actuators while receiving real-time feedback.

An MRI marker system that consists of line markers, an ellipse (E), and a small arc is applied for the robot registration. The points in the MRI can be mapped to the robot system by registering the marker model with its MR image. Therefore, the biopsy point in the MRI is transformed to the point in the robot coordinate. The direction of the needle is determined by the skin entry point, and the insertion depth is calculated by referring to the needle’s depth to the biopsy center.

**Figure 7.**
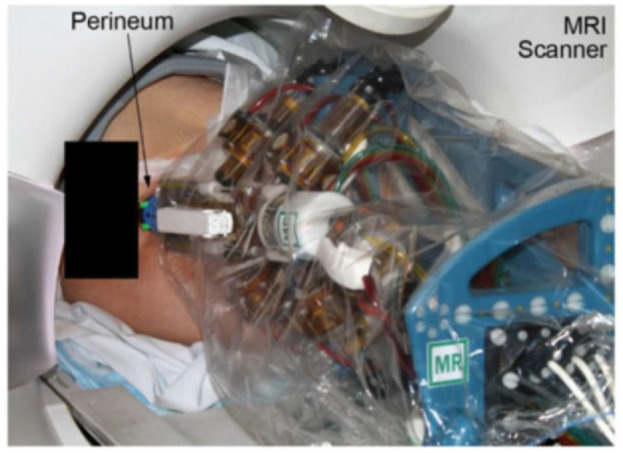
The surgical use of the robot on the patient.^21^

An investigational device exemption (IDE) was applied and was approved by FDA, labeling this device MR safe. In the human test on five subjects (Figure 7), the targeting accuracy of this robot was 2.55 mm, which is acceptable in prostate biopsy.

In summary, the most advanced aspect of this work is its FDA approval. In order to test a robot in a clinical trial, besides the design, numerous other factors such as significant risk (SR) studies, IDE application, sterilization, material biocompatibility, and the robustness of the controller have to be considered. In conclusion, all of these works demonstrate the feasibility of using the prostate intervention robot in MRI, and the clinical applications of the work are beneficial as a guide for other researchers.

### Smart Template

**Figure 8.**
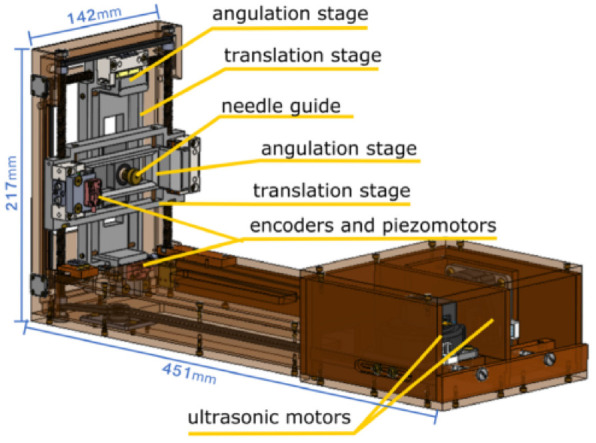
CAD drawing of the Smart Template.^41^

The Smart Templet was developed by Moreira et al. in 2021,^41^ this work was extended from a previous robot from the same group.^48^ The robot has a 2 DOF platform, allowing the needle guide to translate along the horizontal and vertical axes, while an 18-gauge needle can point on the skin through the needle guide (Figure 8). The two translational stages are powered by two ultrasonic motors connecting with brass lead screws, and timing belts are applied to synchronous the movement of both sides. Two extra DOFs are added for the angulations around the two axes, which are driven by two piezoelectric motors connected with optical encoders. The additional DOFs give more flexibility on the path’s selections considering the anatomical structures and insertion point to improve the needle placement accuracy.

A motion controller and four motor drivers placed in the control room are applied to control the two ultrasound motors and piezoelectric motors. A low-power single-board computer (SBC) with a Linux OS running on it is used for the communication between the controller and the user interface. When it is working, the target position set on the user interface is transferred to SBC, then the rotation angle of each motor is calculated in SBC and transferred to the motion controller for each motor.

In the laboratory accuracy test, a 6-DoF optical tracker was applied, showing that the horizontal and vertical positioning accuracies were 0.95 mm, and 0.8 mm, respectively, and the horizontal and vertical angular accuracies were 0.48º and 0.44º. Furthermore, 44 free space insertions were performed with a camera measuring the needle tip location accuracy of 1.3 mm. In addition, the MRI compatibility was measured under different configurations.

The experiment shows that a larger error is observed when the needle is inserted into the lateral side of the prostate. Therefore, the insertion trajectory where the needle is inserted through the bulbospongiosus is proposed to reduce the needle placement error. Sixty-five vivo insertions were performed, and 46 of them were analyzed which showed an average targeting error of 9.1 mm. The bias correction was applied to reduce the needle deviations. Three experiments were conducted, and compared with experiment 1, the error is reduced with bias correction and repeated straight insertions in experiment 2.

In conclusion, the experiment and test of Smart Template show its potential of being used in needle insertions. With a path planning strategy that considers the anatomic structure, needle targeting accuracy can be improved.

## MR-conditional pneumatic actuators

As one of the key components in MRI conditional robots, MR conditional actuators are of great interest to researchers. For an actuator to be MR conditional, all components used in the actuator must be MR conditional, meaning that magnetic and conductive materials are not allowed. Among all the MR conditional actuators, the pneumatic actuators have great potential to be used in the MRI environment because they do not use electricity, and air leakage does not pose a hazard to the clinical environment. Therefore, pneumatic motors become an excellent option for MR safe robots.

This review mainly presents pneumatic actuators designed for the MRI environment in the past 15 years. Pneumatic actuators can be classified into two types based on their working principles and control methods: continuous motor and stepper motor. The characteristics of each actuator, such as size, number of parts, resolution, torque, and speed, are listed in Table 2.

**Table 2.** Comparison of the existing pneumatic stepper motors.

Reference	Year	Group	Number of parts	Size (mm)	Resolution	Torque	Maximumspeed
Tse et al.^49^	2008	ImperialCollege London	~5	Φ44 × 80	-	0.74 N·m witha gearbox	16 rpm
Wei et al.^50^	2016	the University ofHong Kong,	5	203 × 118 × 76	-	30 N·mm	~668 rpm
Sajima et al.^51^	2012	The Universityof Tokyo	~10	Φ30	4.29°	150 N·mmat 0.6 MPa	48 rpm
Chen et al.^52^	2015	The University of Georgia	7	Φ10 × 60	60°	2.4 mN·m	90 rpm
Groenhuis and Stramigioli^53^(T63 motor)	2018	University of Twente	~10	65 × 52 × 36	1 mm	330 N at 0.42 MPa	200 mm/s
Farimani and Misra^54^	2018	University of Twente	>10	30 × 20 × 46	3°	0.14 N·m	800 rpm
Boland et al.^55^	2019	University of Georgia	15	80 × 80 × 80	90°	19 N·mm	2000 rpm
Uzuka et al.^56^ (RS-C)	2009	TOK Bearing Company, Ltd.	~10	Φ50 × 49	3°	2150 mN·m	2 rpm

### Continuous actuators

#### Turbine motor

**Figure 9.**
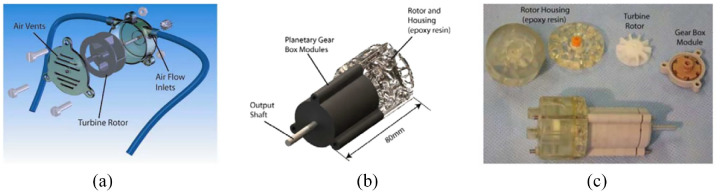
(a) CAD drawing of the motor, (b) assembly of the motor with a gearbox and (c) photo of the motor assembly.^49^

A turbine motor was created by Tse et al. in 2008,^49^ and it was customized and tested to drive an MR conditional robot. As shown in Figure 9, the turbine motor has a simple design and consists of three fabricated parts: housing, rotor, and case. The working principle of this motor is to blow compressed air across the turbine rotor. Two air inlets are placed on the housing, and blowing air from different inlets can drive the motor to rotate in different directions. A planetary gearbox is fitted with the motor to increase the output torque and decrease the speed. Two bearings made of plastics are mounted on the two sides of the rotor to reduce friction and achieve higher speed.

While testing the motor, air pressures ranging from 0.8 to 1.3 bar were applied to the motor. The maximum output torque of 0.74 N·m and the highest speed of 16 r/min were achieved with an airflow of 40 L/min. The compressed air was controlled by solenoid valves, and a pulse width modulated (PWM) signal was used to control the activeness of the solenoid valves. The speed of the motor with respect to different duty cycles of the PWM signal was presented.

**Figure 10.**
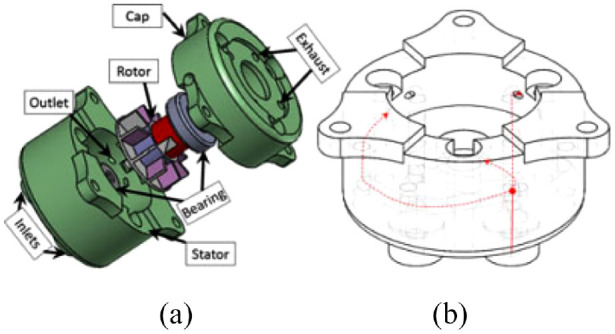
CAD drawing of the motor.^57^

Chen et al. published another paper based on the use of this motor in 2016,^57^ shown in Figure 10. A fiber-optical encoder was built in the motor to provide accurate position and speed control. The motor’s control performance was evaluated when fitted with gearboxes of different ratios. An MRI test showed that this motor had a maximum SNR variation of 5%.

#### Fan motor

**Figure 11.**
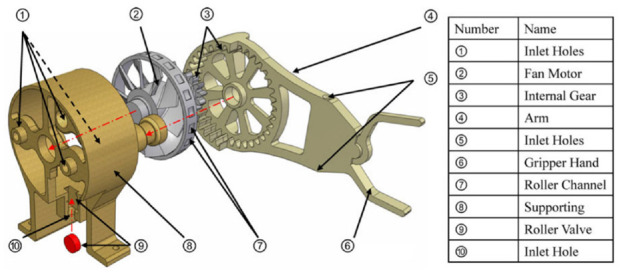
Exploded view of the fan motor.^50^

Wei et al. developed a pneumatic motor based on a fan motor using 3D printing.^50^ The pneumatic motor has an overall size of 203 mm ×118 mm ×76 mm. The mechanism is composed of three components: a fan motor, a supporting frame, and a geared gripper, as shown in Figure 11. The fan is the power source responsible for transferring the power of compressed air into output torque, and the geared gripper is used to drive the load. A roller valve is mounted in the supporting frame. As the blades of the motor have an angle of 45°, the wind blowing on the blades can be transferred to the output torque. Four air inlets are designed for the bi-direct rotation of the motor, and another air hose is connected to the roller valve to control the motion of the roller valve. Twenty channels are made on the outer ring of the blades. By adjusting the air pressure of the roller valve, the engagement of the roller and channels can be controlled. As a result, the motor speed and rotation step can be regulated. Besides, a gear set with a gear ratio of 3:1 is applied on the rotation of the geared gripper to decrease the rotation speed and increase the output torque.

This design allows the pneumatic motor to work under three different pattern modes: natural mode, modulated mode, and stepping mode. In natural mode, no compressed air is applied to the roller valve, so the fan can rotate freely at full speed. In modulated mode, compressed air is applied to the roller valve at different pressures, causing the motor to rotate at different speeds. In stepping mode, compressed air is applied to the roller valve in a pulsed pattern, and the roller is in contact with the channels on the blade synchronously.

In an experiment, the gripper successfully completed simple pick and place actions. It was also demonstrated that angular speed could be regulated by varying the air pressure in the roller valve.

An experiment showed that the starting torque of this motor was 30 N·mm and the maximum velocity was around 70 rad/s. Besides, the fabrication method of 3D printing makes this design easily MR conditional. Overall, the results reflect the potential of the fan motor to be applied in an MR conditional robot.

### Stepper actuators

#### Face gear motor

**Figure 12.**
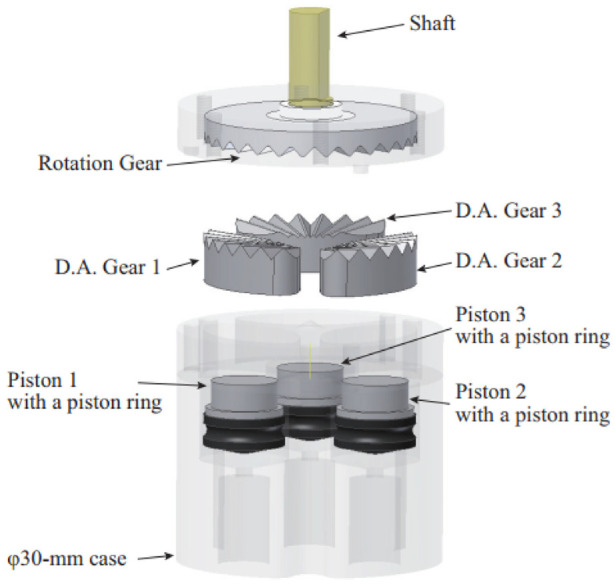
Exploded view of the motor showing the components.^51^

Sajima et al. created a stepper motor in 2010.^51^ This design has a diameter of 30 mm, and as shown in Figure 12, the pneumatic rotation stepping actuator consists of a case, a shaft, a rotation gear (R gear) with 28 teeth, three pistons within syringes, and three direct acting (D.A.) gears.

The working power comes from the pistons, and the motion of the pistons drives the D.A. gears to move linearly. When the D.A. gears are pushed toward the R gear, because of the engagement of the D.A. gears and R gear, the R gear is forced to rotate. Three D.A. gears are equally spaced around the case with an interval of one-third of the pitch. Therefore, one cycle motion of three D.A. gears drives the R gear to rotate through the entire pitch. By controlling the pistons in a bi-directional manner, the shaft connected with the R gear can rotate bi-directionally. As the R gear has 28 teeth, a 360° rotation can be completed with 84 steps.

A performance experiment showed that the motor had a maximum torque of 150 mN·m under 0.6 MPa, and a maximum angular error of 2.1°, which was caused by the gaps between D.A. gears and the case. In an MR safety evaluation, no distortion and no artifacts were found in the MRI images, suggesting that this actuator is MR safe.

The most important advantage of this actuator is its compact size and simple design. The actuator consists of fewer than 10 components, making it one of the simplest MR conditional actuators.

#### Pen motor

**Figure 13.**
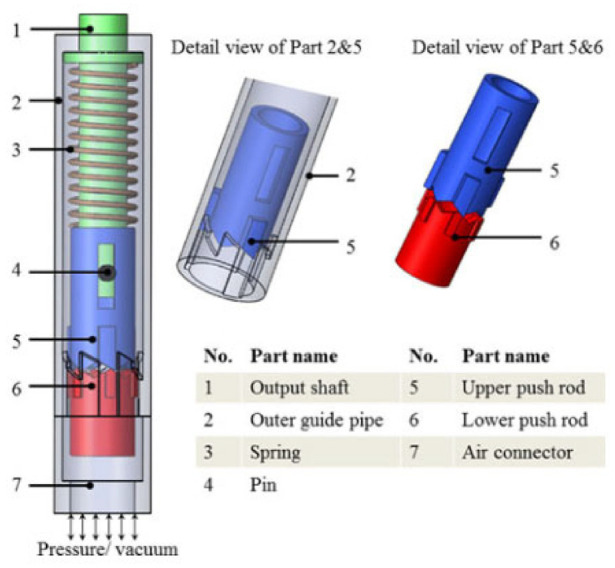
Exploded view of the pen motor.^52^

The Ф10 mm motor developed by Chen et al. (Figure 13) in 2015 is an exceptionally compact motor.^52^ The driving mechanism includes an outer guide pipe, an upper pushrod, and a lower pushrod. At the original position, the upper and lower pushrods are held in place by ridges on the outer guide pipe, and a copper spring is preloaded inside. By pressuring and vacuuming the chamber in the outer guide pipe, the lower push rod can move linearly, driving the output shaft to rotate stepwise. Compressed air is used to push the upper rod to leave the original position and store energy in the spring, and the output torque is generated by the copper spring. Therefore, the motor will produce a greater output torque if the spring has greater stiffness. The air pressure does not affect the torque.

The presented motor has a resolution of 60° and a maximum torque of 2.4 mN·m. An MR compatibility test with a GE 3T MRI scanner showed that it had a maximum artifact width of 3 mm in MR images and a maximum SNR reduction of 2.49%.

The greatest advantage of this motor is its compact size and simple design. The outer diameter is only 10 mm, which makes it possible to be used in some devices with strict size requirements, such as drilling devices and end-effector instruments. However, as the presented motor has only one inlet for pressurized air and vacuum, it can rotate in only one direction, which prevents its application when large torque and bi-directional rotation are needed.

#### Teeth geometry motor

**Figure 14.**
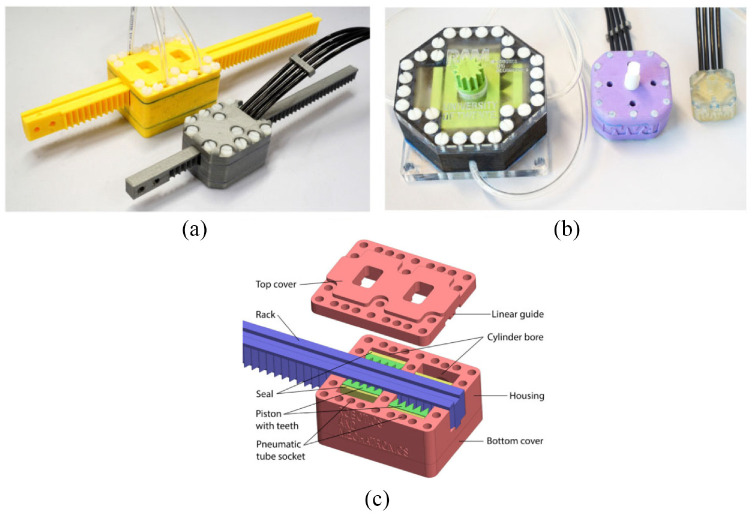
(a) Photo of two linear motors: T49 and T63, (b) photo of three rotatory motors: R25, R44, and R80, and (c) exploded view of T63.^53^

Groenhuis and Stramigioli developed five teeth geometry motors using 3D printed parts and seals.^53^ Three of them (R25, R44, and R80) are rotatory motors, shown in Figure 14(a), and the other two (T49 and T63) are linear motors, shown in Figure 14(b). In this review, T63 (the larger linear motor) is selected as a representative because it has more test results. T49 is a miniaturized version that may be small enough for medical devices.

The motor is made using rapid prototyping and includes two pistons connected with a wedge mechanism, as shown in Figure 14(c). Each piston is connected with two hoses and moves bi-directionally alongside the cylinder to push against the rack. The teeth are located on both sides of the rack, with a linear distance of half-pitch. The adjacent pistons are positioned linearly with a distance of half-pitch as well. Therefore, the activeness of one chamber of the two pistons can push the rack to move a distance of half-pitch, which is the step size of this motor.

An experiment showed that T63 had a maximum output force of 330 N with a mechanical efficiency of 73%. The root mean square error was 0.11 mm, and the repeatability was 0.01 mm. Overshoot and durability were also tested.

In conclusion, the motors developed by Groenhuis et al. at the University of Twente have the strongest output torque and force among all the motors included in this review. The use of teeth introduces a new approach to the design of MR conditional motors and robots. An MR conditional robot for breast biopsy was also developed using five linear teeth geometry motors.^58^

#### PneuAct actuator

**Figure 15.**
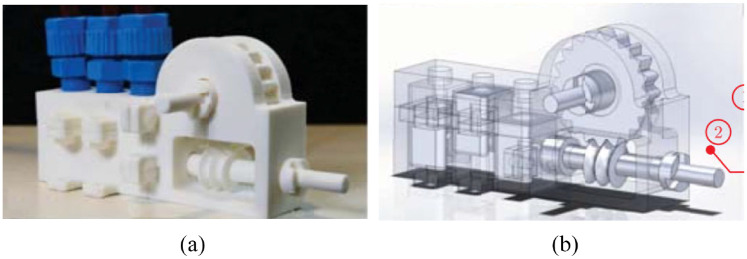
(a) Photo of the motor and (b) CAD drawing of the motor.^54^

Farimani and Misra created a series of pneumatic motors with different dimensions.^54^ The smallest rotational pneumatic stepper motor has the dimensions of 10 ×15 ×28 mm with a volume of 4.6 cm^3^. The motor includes a main body, gearhead, and cylinder-head, as shown in Figure 15. Three pistons are linearly situated in the main body. The gear-head module includes the worm gear and pinion, which can generate a large reduction ratio. When compressed air is introduced into different cylinders sequentially, the pistons drive the crankshaft to rotate. Furthermore, the resolution of the motor is increased by the gear-head module.

The motors can achieve a maximum speed of 800 rpm. MRI tests showed that there were no artifacts or distortions. When a gear-head module with a ratio of 1:40 was used, the maximum torque was 0.14 N·m, and the resolution was 3°.

The main advantage of this motor is that all the components are fabricated using a 3D printer without the use of bearings and seals. Therefore, this motor is inexpensive, so it could be disposable in medical applications. Besides, since the motor is back drivable, it could be used as a passive component in an emergency that requires manually moving the manipulator.

#### Four-cylinder motor

**Figure 16.**
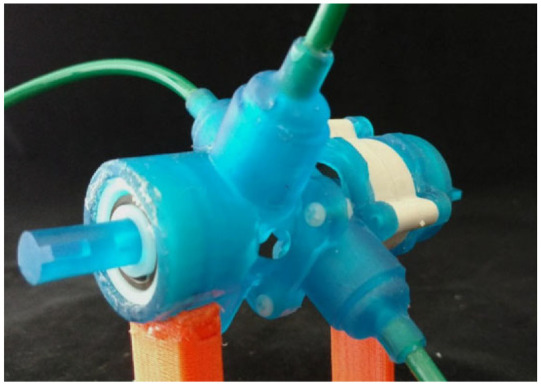
Photo of the four-cylinder motor.^55^

Boland et al. developed a four-cylinder pneumatic motor with the size of 80 mm ×80 mm ×80 mm.^55^ As shown in Figure 16, four cylinders are located in two different planes and are positioned around the center of the motor with a circumference angle of 90°. In Boland et al.,^55^ the components used in the motor are listed, and the output torque with respect to air pressure, crank length, and cylinder radius is presented.

A user-programmable device is used to control the timing of the opening and closing of the valves. The depressurization phase of an air tube before and after each valve closes acts against the motion of the next piston, which limits the maximum speed of the motor. The authors discovered that lowering the “duty cycles,” or adding a small gap between active pressurization periods, could increase the maximum speed of the motor.

When the motor was run during an MRI scan, there was less than 7% of SNR reduction, and no picture artifacts were observed, showing that this motor was MR conditional.

Another experiment showed that the presented motor could achieve a maximum speed of around 2000 rpm and a maximum output torque of 19 N·mm. Moreover, the output torque could be improved by mounting a gearbox.

#### Nutation motor

Although the nutation motors developed by Uzuka et al. in 2009^56^ are made of metal and have not been tested in an MRI scanner, they have an innovative design, and their output torque is much larger than that of conventional electromagnetic motors. Besides, the motors can be driven pneumatically, and their components can easily be made MR conditional, reflecting their great potential to be used in surgical applications. Hence, this design is included in this review.

**Figure 17.**
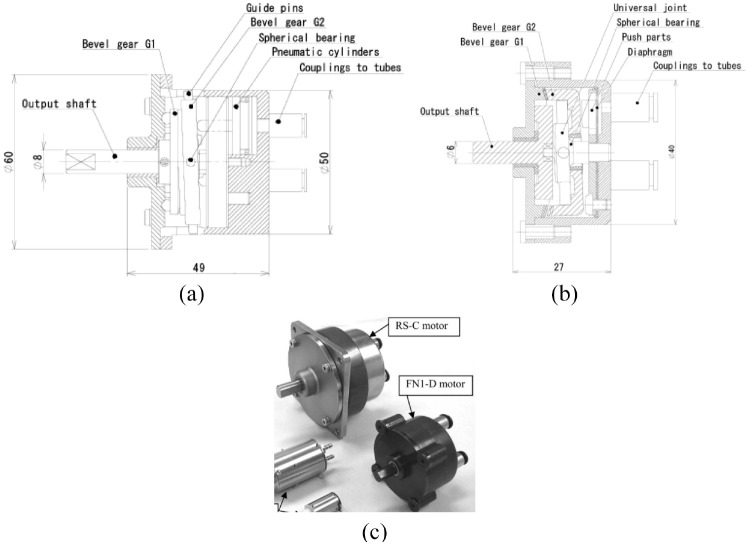
Schematic drawings of nutation motors: (a) RS-C motor, (b) FN1-D motor,^56^ and (c) photo of the RS-C motor and FN1-D motor.

The nutation motor is driven by three pneumatic actuators, and a gear-reduction system is used to convert their action into the rotation of the output shaft. The reduction system has two bevel gears that are engaged with each other. Regarding the RS-C (Figure 17(a)) and RS-D motors, as the teeth numbers of the two bevel gears are different, the linear actuator can drive the nutation motion of bevel gear G1, resulting in the rotation of bevel gear G2. Regarding the FN1-D (Figure 17(b)), FN2-D, and FN2-RD motors, the rotation of bevel gear G2 is prevented, and the output shaft is connected with bevel gear G1. Figure 17(c) shows the photo of the RS-C motor and FN1-D motor. In addition, different motors are obtained by replacing the cylinders with a diaphragm. A diaphragm made of acrylonitrile-butadiene rubber (NBR) is used to minimize the size and reduce the number of components.

The prototype motors have diameters ranging from 10 to 50 mm and lengths ranging from 11.5 to 49 mm. An experiment showed the RS-C motor could achieve an output torque of 2150 mN·m, which is appropriate for applications demanding high output torque. The FN1-D and FN2-D motors are half the length of the other configurations. The diaphragm and FN reduction mechanisms enable miniaturization of the motor, thus making these motors applicable in compact machines. The FN2-RD motor configuration, which acts as a lever, can achieve large output torque by increasing the pressurized areas of the diaphragm.

In summary, various types of motors with the potential to be used in MRI-guided robotic surgeries have been developed.

## Challenges and discussions

This review describes the current development of MR conditional robot systems and actuators. Many studies have demonstrated the possibility of using robots under MRI. Although these are encouraging signs for the future application of MRI-guided robots in clinical environments, some robot systems and actuators have only been tested in animals^26^ or phantoms,^10,14^ and few have seen clinical adoption.^23^ Robot systems suitable for use in MRI are still in their early stages, and there are still some challenges limiting their use in prostate intervention.

One challenge is time efficiency. The use of robot-assisted systems can integrate previously isolated procedures, which can reduce the operation time. The experiment showed the time on individual prostate biopsy of robot-assisted procedure was reduced greatly.^59^ However, in terms of the whole procedure time that was recorded between patients entering and exiting the MRI scanner room, the robot-assisted system had no significant advantages over the manual procedure. Prebiopsy procedures such as robot registration and target planning that are necessary for robot-assisted systems took a long time. This means the procedure time of the robot-assisted approach is not reduced greatly. Considering that the whole procedure has to be performed in the MRI scanner room, the use of MRI-guided procedures would need to be justified in costs and clinical needs.

Another challenge is the variety of manufacturers and models of MRI scanners in hospitals. The different bore diameters, field strengths, and interfaces of different MRI scanners complicate the design of robots to meet the requirements of MRI scanners. Also, different software platforms have to be developed for robots to interface with different MRI scanners.

Due to the strong magnetic fields in the MRI scanner room, all tools and components used in the robots have to be MR conditional. Solutions include using pneumatic actuation, applying optical sensors for feedback, and building the structure of the robot with plastics. Many researchers have worked on developing pneumatic motors used in MRI, but few of these motors have reached the stage of clinical use.

Because of the nonlinear compressibility of air, the major constraint of pneumatic actuators is their poor control. As a result of the compressibility of air, a pneumatic system’s response is relatively slow, so it is difficult to achieve set points rapidly due to the hysteresis. Therefore, the response of a pneumatic motor is not as quick as an electric motor.^60^ This would be a concern in instances where a quick response is required. Moreover, the long hoses running from the MRI control room to the MRI scanner room would aggravate this problem. Although proportional–integral–derivative control and sliding mode control have been applied to control pneumatic motors, the nonlinearity of the dynamic model of the continuous motor would complicate control.^61^ Due to the nonlinear deadband and stick-slip friction of the solenoid valves, precise speed control for the continuous motor running at a low speed is difficult to obtain.^62^

The biopsy and brachytherapy can be performed with real time MRI guidance. Another method is MRI-TRUS Fusion,^63^ in which the MR images are obtained prior to the surgeries, and the pre-interventional MR and the real time ultrasound are spatially aligned during the procedures. This is an advancement over the traditional TRUS technique, it has a simpler operation when compared with MRI guidance and poses a higher detection rate of cancers than the TRUS method.

In addition, the development of single-sided, low-field MR systems like Promaxo makes it ideal for office-based procedures,^64^ which brings greater flexibility to the development and application of MR safe and MR conditional robots. Because of the low magnetic field, few facility upgrades are required. Meanwhile, the workflow for biopsy and brachytherapy can be made more efficient and user-friendly. This simpler and cheaper solution has the potential to expand the clinical applications for both MRI and robots.

## Conclusions

The MR-guided prostate diagnosis, biopsy, and brachytherapy using robot intervention systems becomes a growing topic as prostate cancer becomes one of the most common cancers in men. This review lists the state-of-the-art devices for prostate biopsy and brachytherapy used under the MR environment. Parameters including accuracy, DOF, actuation methods, and MR compatibility are also presented. Besides, the challenges are detailed as well. All of these show the potential of the robot systems to be used in clinical surgeries to provide accurate, reliable operations for diagnosis, biopsy, and brachytherapy. With more and more researchers working on the MR guided robot systems, the development of the MR guided robots would be promising.
